# Addressing Anastomotic Leak After Esophagectomy: Insights from a Specialized Unit

**DOI:** 10.3390/jcm14113694

**Published:** 2025-05-25

**Authors:** Alexandra Triantafyllou, Evgenia Mela, Charalampos Theodoropoulos, Andreas Panagiotis Theodorou, Eleni Kitsou, Konstantinos Saliaris, Sofia Katsila, Konstantinos Kakounis, Tania Triantafyllou, Dimitrios Theodorou

**Affiliations:** 1 First Propaedeutic Department of Surgery, National and Kapodistrian University of Athens, Hippocration General Hospital, 11527 Athens, Greece; alexandra_30fyl@hotmail.com (A.T.); antheo300@gmail.com (A.P.T.); elenimedk@icloud.com (E.K.); konstantinossaliaris@gmail.com (K.S.); t_triantafilou@yahoo.com (T.T.); dimitheod@netscape.net (D.T.); 2 Surgical Department, Tzaneio Hospital, 18536 Athens, Greece; chtheodoropoulos91@gmail.com; 3 Department of Gastroenterology, Hippocration General Hospital, 11527 Athens, Greece; sophie_katsila@hotmail.com (S.K.); kostas.kakounis@gmail.com (K.K.)

**Keywords:** anastomotic leak, esophagectomy, esophageal cancer, esophageal resection, endoscopic therapy

## Abstract

**Background/Objectives:** Anastomotic leakage is one of the most frightening and potentially fatal complications after esophagectomy. The collaboration between the surgical team, interventional gastroenterologists, and radiologists has the potential to improve the hospital stay, as well as morbidity and mortality. The aim of this study is to present our experience and evaluate the results of the multimodal management of anastomotic leak following esophagectomy in our unit. **Methods:** This is a retrospective study analyzing a single referral center’s prospectively maintained database of all patients diagnosed with anastomotic leak between March 2019 and March 2025 using the definition of the Esophageal Complications Consensus Group. The treatment pathways and the patient outcomes are presented. The primary endpoint was 90-day mortality and in-hospital mortality. **Results:** A total of 241 esophageal resections were performed between March 2019 and March 2025. Lymphadenectomy of the mediastinum was performed in 88.4% of the patients. Cervical and intrathoracic anastomosis were performed in 143 (59.3%) and 98 (40.7%) cases, respectively. Twenty-nine patients (12%) with a mean age of 59.1 years developed anastomotic leak. Anastomotic leak occurred in 14.3% of intrathoracic anastomoses and 10.5% of cervical anastomoses. The median day of leak diagnosis was the sixth postoperative day. Leak management involved conservative strategies, wound exploration, endoscopic stent placement or vacuum therapy, drainage of effusions under radiologic guidance, and reoperation. The 90-day and in-hospital mortality rate was 3.4%. No cases of conduit necrosis or mediastinitis were reported. Endoscopic management was employed in 18 patients (62.1%) as a first- or second-line treatment, while reoperation was required in 6 patients (20.7%). The median interval from diagnosis to anastomosis healing was 21 days and the median duration of hospital stay 32 days. The management was successful in 27 patients (93.1%) except for 1 who developed tracheoesophageal fistula and 1 who died due to hemorrhagic complication of anticoagulant treatment. **Conclusions:** Anastomotic leak after esophagectomy is considered a complex, diversified, and morbid clinical entity. The evolving potential of multidisciplinary management encompassing surgical and interventional radiological and endoscopic treatment addresses the mortality rates and heralds a new era of minimizing morbidity.

## 1. Introduction

Anastomotic leak (AL) after esophageal resection remains a dreadful complication of upper gastrointestinal surgery leading to high morbidity rates and affecting functional recovery and oncological results [1]. AL is defined according to the Esophageal Complication Consensus Group (ECCG) as a full-thickness defect involving the esophagus, the anastomosis, the staple line, and/or the conduit irrespective of presentation or method of identification [2]. Despite the major progress in surgical techniques and perioperative care, AL occurs on average in 13.1% of esophagectomies, with a wide range of reported incidences from 3 to 30% [3,4].

AL is a major cause of morbidity and mortality associated with impaired quality of life, prolonged length of hospital and intensive care unit (ICU) stay, increased risk for reoperation, readmissions, increased healthcare costs, and decreased overall survival [4,5,6]. Several risk factors have been analyzed, including both patient-related and technical factors [4,5]. In particular, older age, cardiovascular and metabolic comorbidities, preoperative malnutrition, smoking, and neoadjuvant chemoradiotherapy alongside technical intraoperative variables, such as increased tension and cervical site of the anastomosis, have been reported [7]. The AL clinical spectrum ranges from minor local symptoms without any systemic repercussions to life-threatening sepsis and shock [1,4,5]. Early diagnosis, based on clinical, laboratory, and imaging examinations, is thus paramount for optimal outcomes [4,5].

Effective management of AL requires a multidisciplinary approach including conservative treatment, radiologic or endoscopic procedures, and surgical interventions tailored to the patient’s presentation and severity of symptoms, the time elapsed since esophagectomy, the delay in diagnosis, the size and location of the defect, as well as the extent of contamination [3,4,5]. Although surgery was considered to be the first-line treatment for symptomatic patients, the introduction of endoscopic management has reserved reoperation for severe sepsis, leaks detected within 72 h postsurgery, significant anastomotic disruptions, and uncontained leaks [3]. Endoscopic management with self-expanding metal stent (SEMS) combined with external drainage of pleural effusions constitute the current standard of care for symptomatic contained leaks.

Alternative endoscopic options include endoclips, tissue sealants, suturing systems, and endoscopic vacuum therapy (EVT) [3,5]. The latter, since it was introduced in 2008 by Weidenhagen et al. for the treatment of AL after anterior rectal resection, has revolutionized foregut surgery by highlighting a shift towards non-surgical interventions for AL [8,9].

Addressing AL following esophagectomy is challenging, and standardized treatment protocols and consensus are lacking. The aim of this study is to present and evaluate the therapeutic approaches and the outcomes of patients with AL after esophageal resections during a 6-year period in a tertiary esophageal surgery reference center.

## 2. Materials and Methods

### 2.1. Patient Population

This retrospective study was based on a prospectively collected institutional database of all patients who underwent esophageal resection at the specialized unit of Hippocration University Hospital, Greece, which constitutes a national referral center for upper gastrointestinal surgery, from March 2019 to March 2025. All patients who developed AL after esophagectomy as defined by the ECCG were included in this study [1]. Τhere were no exclusion criteria. Patient demographic and clinical data were collected from the institutional database and included the following: (i) age, gender, body mass index (BMI), American Society of Anesthesiology (ASA) score, comorbidities; (ii) indication for esophageal resection; (iii) neoadjuvant therapy; (iv) type of surgery and site of anastomosis; and (v) details of postoperative complication management and outcomes. Postoperative complications were reported according to the Clavien–Dindo (CD) classification of surgical complications [10]. This study received Institutional Review Board (IRB) approval from the Hippocration General Hospital of Athens, under approval number 10683/07-06-24, dated 7 June 2024. Written informed consent covering operative risks and the use of their data for scientific purposes was obtained from all patients.

### 2.2. Surgical Technique

All procedures in the Upper Gastrointestinal Surgery Unit were performed by two specialized surgeons. Following resection, the site of the anastomosis was either cervical or intrathoracic. For the cervical anastomosis, a hand-sewn, end-to-side technique using PDS 3/0 (Ethicon/Johnson & Johnson MedTech, Cincinnati, OH, USA) simple interrupted sutures was performed. For the intrathoracic anastomosis, a semi-stapled, end-to-side technique with a laparoscopic linear 60 mm stapler and blue reloads followed by hand-sewing of the defect using a barbed 3/0 running V-Loc (Medtronic/Covidien; Minneapolis, MN, USA) was performed. Since January 2023, a transition was made to a totally hand-sewn, two-layer, end-to-side technique with barbed 3/0 running V-Loc (Medtronic/Covidien; Minneapolis, MN, USA) including four to five tension-release stitches to minimize the traction on the anastomosis. In January 2025, a transition was made from V-Loc to 3/0 running Stratafix (Ethicon/Johnson & Johnson MedTech, Cincinnati, OH, USA).

### 2.3. Anastomotic Leak Diagnosis and Management

During the postoperative period, patients did not routinely undergo radiologic examination for assessing the integrity of the anastomosis. Upon clinical suspicion of AL and a rise in inflammatory blood markers such as leukocytes and CRP, all patients were treated with a nil per os regimen and received broad-spectrum intravenous antibiotics, including antifungals. Ιf needed, a computed tomography (CT) scan was performed with administration of 1.5 mL/kg intravenous contrast agent and 15 mL of oral water-soluble contrast agent. All CT images were reviewed by an experienced radiologist. Extraluminal leak of contrast and local presence of air at the level of the anastomosis were considered indicative of AL. In addition, diagnostic paracentesis with biochemical examination of the fluid for amylase was utilized selectively in patients with inconclusive radiologic findings. Upper gastrointestinal endoscopy was performed if required to confirm the diagnosis, assess the viability of the conduit, and treat AL when indicated. Further treatment decisions were made by a multidisciplinary team depending on the patient’s clinical condition and on availability, expertise, and costs.

If the leak was considered suitable for endoscopic treatment, either SEMS or intraluminal vacuum therapy (B. Braun Melsungen AG, Melsungen, Germany) was applied. In particular, Esophageal Controlled-Release Fully Covered Metal Stents of 20–25 mm × 10 cm (EVO-FC-20-25-10-E) or 20–25 mm × 12 cm (EVO-FC-20-25-12-E) (COOK Medical LLC, Bloomington, IN, USA) were utilized, and in cases where stent fixation was necessary, through-the-scope (TTS) clips with an Instinct Plus™ Endoscopic Clipping Device (COOK Medical LLC, Bloomington, IN, USA) were applied. When significant fluid collections were present, drainage was managed through secondary surgical or interventional drains. Nutritional support after stent deployment was determined by the dietitian and surgeon. Unless specified otherwise, patients were initially placed on a liquid diet, which was gradually advanced as tolerated, with parenteral nutrition initiated to meet daily nutritional requirements. In cases of early leak (<72 h) with severe sepsis, complete anastomotic dehiscence, or failure of endoscopic treatment, surgical treatment was implemented. After hospital discharge, routine endoscopic reevaluation was scheduled approximately 6 to 8 weeks later depending on the patient’s condition and the size of the leakage, aiming for stent removal.

### 2.4. Outcomes

The primary outcomes were in-hospital and 90-day mortality. Secondary outcomes were considered the time to anastomosis healing, the days between esophagectomy and diagnosis of AL, length of hospital stay, ICU admission, readmission rate, and number of interventions required. Additionally, complications following the endoscopic intervention were documented.

### 2.5. Statistical Analysis

For the present study, descriptive analysis was mainly opted for. Categorical data are demonstrated as count with percentage rate, while continuous data are presented as mean with standard deviation (SD) or median with interquartile range (IQR) based on the type of data. Mortality rates were calculated as percentages with corresponding 95% confidence intervals (CIs) using the Clopper–Pearson exact method for binomial data. Categorical variables were compared using the Chi-square test. All analyses were performed using R Statistical Software (version 4.4.2), and a *p*-value of <0.05 was considered to be statistically significant.

## 3. Results

### 3.1. Patient Demographics

During the study period, a total of 241 esophageal resections were performed in our unit, after which 29 patients developed AL (12%) and were included in this study. Transthoracic esophagectomy was the most common procedure with 213 cases (88.4%). Cervical and intrathoracic anastomoses were performed in 143 (59.3%) and 98 (40.7%) cases, respectively. Anastomotic leak occurred in 14 patients (14.3%) with intrathoracic anastomoses and 15 patients (10.5%) with cervical anastomoses. The odds of developing AL were 21% lower in the cervical anastomosis group compared to the intrathoracic group, yet this difference was not statistically significant (95% CI 0.32–1.53, *p* = 0.374). Table 1 consolidates patients’ characteristics. The majority of patients were males (25 males and 4 females), and the mean age was 59.1 years. The median BMI value was estimated as 28 kg/m^2^, with most patients being classified as ASA II (82.8%). Regarding the indication for esophagectomy, 27 (93.1%) patients had malignant while 2 (6.9%) had benign conditions. In particular, 25 (86.2%) patients were diagnosed with adenocarcinoma located on the lower third of the esophagus or the esophagogastric junction, while 1 (3.45%) patient was diagnosed with squamous cell carcinoma and 1 (3.45%) with esophageal leiomyosarcoma. Additionally, one (3.45%) patient had a traumatic etiology related to Boerhaave syndrome with delayed repair and another one (3.45%) due to caustic ingestion.

Of those with malignant indication, 23 (85.2%) received neoadjuvant chemotherapy and 1 (3.7%) received neoadjuvant chemoradiotherapy. Only three patients exhibited positive stress echocardiogram for ischemia and required more extensive perioperative monitoring. Furthermore, 23 patients (79.3%) had no arterial calcifications.

### 3.2. Clinical Presentation and Diagnosis

Among the 29 patients with AL, 6 (20.7%) developed early leakage within 72 h in their postoperative course. The median day of AL diagnosis was the sixth postoperative day (IQR 4–9 days). Twenty-one patients (72.4%) developed fever, fifteen patients (51.7%) experienced tachycardia, and only two (6.9%) experienced tachypnea. All patients with cervical AL developed local surgical wound findings. Twenty-two patients (75.9%) exhibited changes in their abdominal drains or chest tubes, such as alterations in the amount or color of the drainage. Additionally, drain biochemical testing was performed in nine patients (31%) with clinical suspicion of leakage, with all of them being positive for amylase (>3 ULN). AL was diagnosed with a CT scan in 24 out of 29 patients (82.8%), with 14 (48.3%) patients also undergoing upper gastrointestinal endoscopy. In the remaining five patients (17.2%) with cervical anastomosis, the diagnosis was based solely on clinical image and laboratory findings. At the time of AL diagnosis, the mean serum level of CRP was 195.14 (SD 106.89), while the mean leukocyte count was 11,531 (SD 4902).

### 3.3. Management of AL

**Figure 1:**
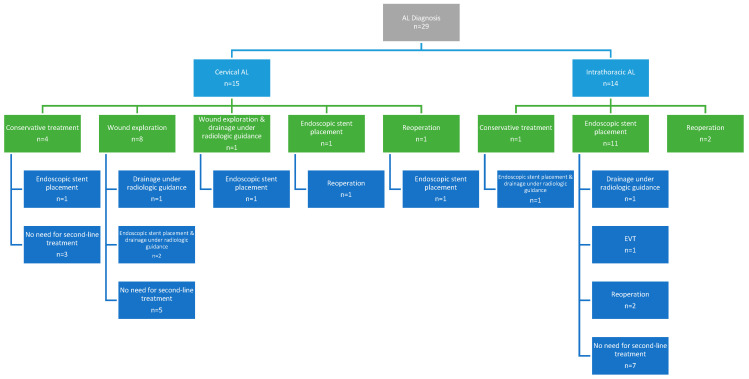
Anastomotic leak management.

The predominant number of patients (*n* = 23, 79.3%) received non-operative management. The management of AL is summarized in Figure 1. In particular, conservative management with intravenous antibiotic therapy and nil per os regimen was employed as a first-line treatment in five patients (17.2%). In cervical ALs, nine patients (60%) required wound exploration and drainage. Endoscopy was performed to assess the integrity of the anastomosis and to place a SEM in 12 patients (41.4%) as a first-line and in 6 patients (20.7%) as a second-line treatment. Among them, six patients (33.3%) had a cervical anastomosis site and leakage. Of the 18 patients who underwent endoscopic stent placement, stent fixation with clips was necessary in 10 patients (55.6%). In terms of stent-related complications, six patients (33.3%) experienced stent migration, with one additionally experiencing bleeding. In addition, in two individuals (11.1%), the stent’s diameter was less than that of the gastric conduit’s lumen, resulting in ongoing leakage. Consequently, a second stent (stent-in-stent) was employed in the two latter patients, as well as three patients with stent migration. One patient (3.4%) required EVT as a second-line treatment following the clinical failure of the stent to control the leakage. The patient required four EVT changes and achieved endoscopic healing of the defect within 15 days of therapy. The median number of endoscopies per patient was 2.5 (IQR 2–3). Drainage of effusions under radiologic guidance was performed as an adjuvant therapy in six patients (20.7%). In this context, three patients (10.3%) underwent reoperation as primary and three (10.3%) as secondary management. Out of the six patients, all (100%) underwent drainage of collections, three patients (50%) underwent lung decortication and one (16.7%) feeding jejunostomy placement. Two patients (33.3%), who experienced early postoperative non-contained leakage, underwent suture reinforcement of the anastomosis along with drainage. The majority of patients were treated non-surgically, and leaks were classified as type I in 3 patients (10.3%) and type II in 20 patients (69%) according to the ECCG grading system. The remaining six patients (20.7%) required reoperation and were considered as type III leaks. No ischemia or necrosis of the conduit was documented, and there were no cases of mediastinitis. Overall, first-line treatment was successful in 58.6% of patients, with 12 patients (41.4%) requiring a second-line treatment.

### 3.4. Clinical Outcomes

The median total length of hospital stay was 32 days (IQR 22–45), with four patients (13.8%) necessitating readmission to the ICU, among whom three had cervical ALs. The readmission rate to the hospital was 37.9% (*n* = 11), with four patients having intrathoracic ALs. The primary outcome of in-hospital and 90-day mortality rate was 3.4% (*n* = 1/29; 95% CI: 0.09–17.72%). This patient with non-contained intrathoracic leak who underwent reoperation on the first postoperative day and following that was diagnosed with pulmonary embolism died due to hemorrhagic complication of anticoagulant treatment. The median interval from AL diagnosis to healing was 21 days (IQR 10–38). A tracheoesophageal fistula occurred in one patient (3.4%) with intrathoracic AL following endoscopic stent treatment, 59 days following esophagectomy. The multimodal management was thus successful in 27 patients (93.1%). Strictures were diagnosed in five patients following a median interval of 63 days (IQR 55–97) postesophagectomy. All patients had cervical anastomoses and were treated with endoscopic dilatations with a median of three dilatations per patient (IQR 3-13). The aforementioned findings are summarized in Table 2.

## 4. Discussion

The present study evaluated the treatment strategy and outcomes of AL secondary to esophagectomy at a tertiary referral center. We recorded an AL rate of 12%, which is consistent with the expected range reported in the literature [3,4]. According to the ECCG, the severity grade of AL is defined by the treatment strategy applied (conservative, endoscopic, surgical) [1].

Concerning risk factors, different surgical anastomotic techniques alongside a range of patient-related conditions and oncological and perioperative variables are considered to influence the AL rates following esophagectomy and constitute an area for future investigation [11]. Patient-related factors include older age, male gender, obesity, tobacco use, nutritional depletion, neoadjuvant radiotherapy, and comorbidities impairing microvascular perfusion. In this context, the observed low prevalence of arterial calcifications (20.7%), which represent an emerging risk factor for AL and a surrogate marker of compromised tissue perfusion, may partially account for the outcomes in our cohort [12]. Additionally, perioperative factors, comprising prolonged mechanical ventilation, intraoperative hypotension, fluid management, catecholamine use, and the need for blood transfusion, further influence the likelihood of AL [7,11]. Intraoperative anesthesiology monitoring and practices are, thus, paramount in optimizing modifiable risk factors, and in our institution, standardized anesthetic protocols incorporate immediate postoperative extubation, protective ventilation settings, and meticulous intraoperative fluid management to support this objective [4,7].

Technical elements linked to the incidence of AL include the location and technique of the anastomosis as well as the employment of pedicled omental flaps [11]. The incidence of AL based on anastomotic site in our cohort was 14.3% for intrathoracic anastomosis and 10.5% for cervical anastomosis, which does not align with the understanding that cervical anastomosis is a risk factor for AL. However, the difference in AL incidence among the two groups was not statistically significant (95% CI 0.32–1.53, *p* = 0.374) [7,11,13]. Despite this, a recent meta-analysis found no difference in postoperative mortality between intrathoracic or cervical anastomosis [14]. Regarding intrathoracic anastomoses, a recent meta-analysis found significantly higher risk for AL in hand-sewn and side-to-side linear-stapled anastomoses compared to circular-stapled [15]. However, the comparatively increased AL rate of 14.3% for intrathoracic anastomoses may be partially attributable to the adoption of new techniques, specifically linear stapling within the time period of 2021–2023, followed by a transition to hand-sewn anastomosis. In this context, in pursuit of reducing the risk and/or the severity of AL, intraoperative perfusion monitoring for assessing gastric tube viability with indocyanine green (ICG) fluorescence angiography as well as ischemic preconditioning with partial gastric devascularization prior to esophagectomy are emerging approaches with encouraging results, especially in high-cardiovascular-risk patients [7,13]. Furthermore, early diagnosis of AL based on biochemical and imaging tests improves prognosis and facilitates early initiation of conservative measures, potentially avoiding the need for invasive management [16].

AL after esophagectomy significantly affects postoperative morbidity and mortality, thus compromising the quality of life, the long-term recovery, and the oncologic outcomes [5,11]. Formerly documented success rates in the literature of different treatment modalities for AL vary from 50 to 100% [14]. In this context, two recent multicenter studies involving 319 and 1508 patients with AL exhibited 90-day mortality rates of 12% and 11.7%, respectively [5,17]. Low failure-to-rescue rates have been correlated with large-volume centers with a wider range of therapeutic modalities, reduced leak severity, and need for ICU stay [18]. In the current cohort, the 3.4% 90-day mortality rate is substantially less than the stated literature rates. In addition to the low mortality rates, leakage was effectively managed in 27 patients (93.1%).

The management of AL has altered from extensive surgery to conservative and endoscopic approaches. Decision-making regarding management is influenced by the clinical manifestation, anastomotic location, severity of symptoms, timing of diagnosis, and available expertise [13]. Conservative management, including nil per mouth for an average duration of 1–3 weeks, total parenteral nutrition, and antibiotic therapy is reserved for asymptomatic or minimally symptomatic patients with contained cervical leaks [4,13]. In case of non-spontaneously drained wound abscess in cervical incision, reopening and rinsing with isotonic solution is indicated for adequate external wound drainage. In our series, four patients (26.7%) with cervical leakage were managed conservatively in the first-line of treatment, while nine patients (60%) required opening of the cervical wound. All the patients had contained cervical leaks without intrathoracic involvement. Although in the worldwide literature intrathoracic anastomoses are linked with lower leakage incidences, therapy differs due to the greater risk of life-threatening mediastinitis and sepsis [13,14].

The advent and continuing advancement of endoscopic treatment has provided an alternative to surgical management for symptomatic patients not manageable with conservative strategies [13]. Numerous established endoscopic treatments for AL are available to restore GI continuity [19]. Endoscopic stents are widely employed in managing ALs with reported overall success rates ranging from 44% to 88%, and stent migration and tissue overgrowth constitute the two main adverse effects [14,20]. In the current series, 85.7% of patients with intrathoracic AL and 40% with cervical AL underwent endoscopic stent placement, among which 55% required clip fixation. The most common stent-related complication was stent migration, documented in six patients (33.3%), which is consistent with the rates reported in the literature, typically ranging from 16% to 62% depending on stent type, location, and fixation technique [21,22,23].

Historically, SEMS was considered the gold standard for treating intrathoracic esophageal leaks. However, the emergence of EVT and its outcomes have challenged this assumption [21,22,23]. The vacuum-assisted closure therapy constitutes now a well-established technique that promotes tissue healing by removing secretions, reducing bacterial proliferation, and enhancing microcirculation and granulation tissue proliferation [9,13]. In a meta-analysis of 338 patients, EVT outperformed SEMS with a significantly greater closure rate of 85% as opposed to 65% [23]. In our practice, SEMS was the standard endoscopic procedure, with EVT utilized as second-line therapy in only one patient with persistent leakage and failure of SEMS. The patient required four sponge changes and a 15-day therapy duration to achieve endoscopic defect healing.

The endoscopic treatment landscape for AL after esophagectomy is constantly advancing. Novel endoscopic approaches encompass TSCs, over-the-scope clips (OTSCs), tissue sealants, and endoscopic suturing. In our unit, none of the patients were treated with these techniques, which corresponds to the lack of large-scale studies on their effectiveness and safety [13,18]. However, favorable outcomes for the stent-over-sponge technique have been recently documented, particularly for complex leaks, combining the SEMS’s advantages for lumen patency and EVT’s for secretion removal and granulation tissue growth [13,18,24]. Therefore, future directions are emerging in the research landscape of AL management, not only in the prediction and prevention of AL through advanced imaging and perfusion assessment but also in endoscopic treatment, with particular focus on further validation through prospective studies of the stent-over-sponge technique to optimize outcomes and support individualized therapy.

Surgical intervention is currently reserved for early leaks within the first 72 h since they are usually non-contained and linked with technical errors or conduit necrosis leading thus to rapid clinical deterioration, for severe sepsis, and for failure of conservative or endoscopic approaches [11,14]. The surgical approach should be individualized and depends on the extent of the anastomotic defect, the degree of containment, the presence of ischemia, and the patient’s condition [14,25]. In our series, reoperation was required for six patients, three as a primary treatment and three as a secondary treatment. Only two patients necessitated resuturing of the anastomotic defect, while six patients underwent drainage and three patients underwent debridement through a thoracoscopic approach.

## 5. Limitations

Among the limitations of this study are its retrospective methodology and the small sample of patients from a single institution, which impacts generalizability. In addition, it included individuals who did not undergo a uniform surgical approach, encompassing both transhiatal and transthoracic esophagectomies, which may have introduced variability in outcomes. Additionally, the treatment algorithm evolved over the study period. In particular, EVT was introduced in our unit in 2024; therefore, only a single patient could undergo this treatment. Therefore, these findings underscore the need for future prospective multicenter studies to validate and expand upon these results.

## 6. Conclusions

AL after esophagectomy is a challenging and diverse clinical entity associated with increased morbidity and mortality. Early diagnosis remains paramount to address the morbidity rates. In the era of minimally invasive and endoscopic techniques, treatment algorithms have shifted from surgery to conservative and less invasive strategies, with growing implementation of the endoscopic approach. This study exhibited that multimodal management of AL was effective in 93.1% of patients and the 90-day mortality rate accounted for 3.4%. Thus, the evolving promise of multidisciplinary management, involving interventional radiological and endoscopic treatment along with surgery as the last resort, has addressed mortality and ushers in a new era of reducing morbidity and optimizing patients’ quality of life.

## Figures and Tables

**Table 1:** Patient characteristics.

Variables	*n* = 29
Age (mean, SD)	59.1 (12.2)
BMI (median, IQR)	28 (26–29.7)
Gender	
Male	25 (86.2)
Female	4 (13.8)
ASA score	
1	1 (3.4)
2	24 (82.8)
3	4 (13.8)
4	0 (0)
Charlson Comorbidity Index (median, IQR)	4 (3–4)
Indication for esophagectomy	
Malignancy	27 (93.1)
Benign	2 (6.9)
Neoadjuvant therapy	
None	5 (17.3)
CT	23 (79.3)
CRT	1 (3.4)
Type of anastomosis	
Esophagogastric	27 (93.1)
Esophagocolonic	2 (6.9)
Site of anastomosis	
Cervical	15 (51.7)
Intrathoracic	14 (48.3)
Time period from surgery to leakage diagnosis, days (median, IQR)	6 (4–9)
ECCG AL grade	
Type I	3 (10.3)
Type II	20 (69)
Type III	6 (20.7)
Clavien–Dindo Classification	
2	3 (10.3)
3	22 (76)
4	3 (10.3)
5	1 (3.4)
Pathologic T stage	
pT0	5 (18.5)
pT1	5 (18.5)
pT2	5 (18.5)
pT3	10 (37)
pT4	2 (7.5)
Pathologic N stage	
pN0	15 (55.5)
pN1	3 (11.1)
pN2	5 (18.5)
pN3	4 (14.9)
Pathologic M stage	
pM0	27 (100)
pM1	0 (0)

SD, standard deviation; IQR, interquartile range; ASA score, American Society of Anesthesiology score; CT, chemotherapy; CRT, chemoradiotherapy; ECCG, Esophagectomy Complications Consensus Group; AL, anastomotic leakage. Values are expressed as *n* (%), median (IQR), or mean (SD).

**Table 2:** Clinical outcomes.

Variables	*n* = 29
In-hospital mortality	1 (3.4%)
90-day mortality	1 (3.4%)
Total length of hospital stay, days (median, IQR)	32 (22–45)
Readmission to the ICU	4 (13.8%)
Readmission to the hospital	11 (37.9%)
Interval from diagnosis to defect healing, days (median, IQR)	21 (10–38)
Tracheoesophageal fistula	1 (3.4%)
Strictures	5 (17.2%)

Values are expressed as *n* (%), median (IQR).

## Data Availability

The data presented in this study are available on request from the corresponding author. The data are not publicly available due to the department’s and IRB’s policy.

## Abbreviations

The following abbreviations are used in this manuscript:

AL	Anastomotic leak
ECCG	Esophageal Complication Consensus Group
ICU	Intensive care unit
SEMS	Self-expanding metal stent
EVT	Endoscopic vacuum therapy
BMI	Body mass index
ASA	American Society of Anesthesiology
CD	Clavien–Dindo
IRB	Institutional Review Board
CT	Computed tomography
TTS	Through-the-scope
SD	Standard deviation
IQR	Interquartile range
OTSCs	Over-the-scope clips
